# Nutritional Knowledge, Attitudes, and Practices of Women Living with HIV in Eastern Uganda

**DOI:** 10.3329/jhpn.v28i2.4890

**Published:** 2010-04

**Authors:** John Bukusuba, Joyce K. Kikafunda, Roger G. Whitehead

**Affiliations:** ^1^ Department of Food Science and Technology, Makerere University, PO Box 7062, Kampala, Uganda; ^2^ Church End, Weston Colville, Cambridge CB1 5PE, UK

**Keywords:** Acquired immunodeficiency syndrome, Cross-sectional studies, Diet, Human immunodeficiency virus, Knowledge, attitudes, practices, Nutrition, Uganda

## Abstract

HIV and AIDS have posed various medical, nutritional, social and economic problems, female-headed households being the most affected. Poor nutritional knowledge and dietary practices common among the most affected households significantly contribute to the rapid progression of HIV. However, very little data exist concerning these aspects of nutrition among women living with HIV and AIDS in resource-limited settings, such as Uganda. The aim of the study was to investigate the gaps in nutritional knowledge, attitudes, and practices and their relationship with sociodemographic characteristics in an urban population of women living with HIV and AIDS in Uganda. In total, 133 women living with HIV were interviewed using a pretested questionnaire. Most (89.5%) women reported being trained on the importance of nutrition for people living with HIV and AIDS (PLWHA) and believed that it is very important to consume a balanced diet (99.5%). On the contrary, only 21.8% consumed at least three meals per day and 39.8% at least six food-groups. They also reported higher dependency on starchy staples while foods of animal origin and fruits that play vital immunity and protective roles were inadequately consumed. Results of bivariate analysis indicated that consumption of a diversified diet was significantly associated with access to food-aid (p=0.006), possibly because access to food-aid reportedly enhances the ability of the household to access other food items. However, much is still needed to understand the drug-food interaction and dietary diversification and enhance proper dietary practices through sustainable projects that ensure increased access to food. Support groups of the PLWHA are a good vehicle for communication of nutrition information and implementation of nutrition-related projects.

## INTRODUCTION

Uganda's policies are credited with helping to bring the HIV prevalence among adults down from around 18.5% in the early 1990s to about 6.7% in 2005 (1). However, a higher rate of HIV prevalence was still registered in the urban setting (10.2%) compared to rural areas (5.7%). The prevalence, disaggregated by gender and urban-rural dimensions, showed a higher infection (12.8%) among urban women than among their rural counterparts (6.7%) (2). The urban setting of Jinja district in Eastern Uganda is among those with the reportedly highest rates—10% of HIV prevalence among adults (3, 4). The epidemic has devastated many sectors in the country, including agricultural production, consequently the ability of households and communities to secure adequate food (5). The report showed that HIV and AIDS have also posed various medical, nutritional, social and economic problems nationwide, female-headed households being the most affected.

Addressing gaps in nutrition among people living with HIV and AIDS (PLWHA) is essential because nutrition plays a vital role in the care and management of HIV and AIDS as it is intrinsically linked to immune function (6–8). However, scanty information exists on studies of these linkages in Uganda. Research has shown that both macronutrient and micronutrient deficiencies contribute to immune dysfunction and can lead to progression of disease (6–9). However, consumption of proper nutrients, which can be enhanced by knowledge of importance of good nutrition for the PLWHA and proper dietary practices, can support an already-compromised immune system (9, 10). This study was, therefore, undertaken to assess the gaps in nutritional knowledge, attitudes, and dietary practices of women living with HIV and their interactions with sociodemographic characteristics, so as to report on some effective ways to improve the nutritional management of HIV and AIDS in this vulnerable group.

## MATERIALS AND METHODS

### Study population and design

Data for this cross-sectional study were collected from 133 women living with HIV in Jinja district, Eastern Uganda. The women receive services for HIV from The AIDS Support Organization (TASO), Jinja Network of People Living with HIV and AIDS (JINNET+) and/or AIDS Orphans Education Trust (AOET). Participants were eligible for the study if they were residents of Jinja municipality and registered in any of these three HIV service organizations. A list was made comprising all eligible women living with HIV to develop a sampling frame. Random selection from the list was then done to establish the sample size of 133 women for the study. A pretested questionnaire was used for collecting data in the homes of the participants to obtain sociodemographic information, health characteristics of the participants and the household members, nutrition knowledge, attitudes, and dietary practices.

### Nutritional knowledge, attitudes, and practices

#### Nutritional knowledge

Four questions were used for assessing the nutritional knowledge of the participants. These included: (a) participation in training on nutrition for the PLWHA, (b) correct definition of good nutrition, (c) knowledge of dietary recommendations for the PLWHA, and (d) knowledge of the relationships between diet and disease, including the consequences of poor nutrition when living with HIV and AIDS.

#### Attitude on nutrition

The participants were asked four questions to assess their attitudes towards nutrition recommendations for the PLWHA. These included their thoughts about the importance and usefulness of (a) eating various foods, (b) consumption of fruits and vegetables, (c) increasing meal frequency, and (d) consumption of special diets.

#### Dietary practices

Five aspects of dietary consumption patterns were used for assessing the dietary practices of the participants: (a) number of meals consumed in the preceding 24 hours to the survey, and (b) number of food-groups consumed (9). The foods reportedly consumed in the preceding 24-hour recall were grouped in 12 food-groups: cereals, roots/tubers, legumes, milk/milk products, fish, poultry, meat, eggs, fruits, vegetables, oils/fats, and sugar/honey (5, 11), (c) reported consumption of special diets, (d) portioning of meals within the household, and (e) food taboos.

Based on the number of meals consumed, the percentage of households eating less than three meals per day was determined. The frequency of meal has been shown to serve as a proxy indicator of consumption of macronutrients (11, 12). Households were also categorized into those that consumed an adequate diet of good quality (≥6 food-groups) or inadequate (<6 food-groups) based on the total number of food-groups (13, 14). Dietary diversity measured by the number of food-groups consumed has been shown to be a potential ‘proxy’ indicator of adequacy of nutrients (15, 16).

### Access to food-aid

Selected vulnerable households receive food-aid through the Title II HIV/AIDS Life Initiative. The Agricultural Cooperative Development International/Volunteers Overseas Cooperative Assistance (ACDI/VOCA) programme funded by United States Agency for International Development was started in 2002. Its primary objective is to bridge the gap of food insecurity among the PLWHA and improve their quality of life. In Jinja district, the programme has been implemented through TASO. Beneficiary households receive monthly rations of corn-soy blend (CSB) and vegetable oil according to the size of the household.

### Statistical analysis

The SPSS software (version 12) was used for data-cleaning and analyses. Bivariate analyses (chi-square) were used for determining the interactions between the nutritional knowledge, attitudes, and dietary practices of the participants and their sociodemographic characteristics.

### Ethical approval

The Department of Food Science and Technology of the Makerere University, Uganda National Council for Science and Technology, and the research committee of TASO approved the study. Consent of the participants was obtained before recruitment.

## RESULTS

### Sociodemographic characteristics of study participants

The dominant tribe in the district is Basoga. The large majority (72.2%) of the participants were of Basoga tribe, and the average size of household was seven. The mean age of the participants was 34 years, and the majority (60.2%) were widows who were heads of their own households and main breadwinners. The majority (50.4%) of the participants were neither employed nor engaged in any form of business (Table 1). The highest educational level attained by most study participants was primary (54.9%).

**Table 1.** Sociodemographic characteristics of study participants (n=133)

Variable	No.	%
Tribe		
Musoga	96	72.2
Muganda	17	12.8
Other	20	15.0
Marital status		
Never married	7	5.3
Divorced/separated	10	7.5
Married	36	27.1
Widowed	80	60.2
Occupation		
Neither employed nor doing business	67	50.4
Informal business	53	39.8
Formal business	8	6.0
Salaried employment	5	3.8
Level of education		
No formal schooling	17	12.8
Primary school	73	54.9
Secondary+	43	32.3

### Health characteristics of study participants and their households

The large majority (73%) of the participants had been living with HIV for more than one year, and 72.2% sought healthcare immediately after the diagnosis of HIV mainly from HIV service organizations in the district (Table 2). According to the national protocols for administering antiretrovirals (ARVs), 18% of the participants were on ARVs while the remaining participants were either on therapy for tuberculosis (TB) or their CD4 count was still in the normal range. Antiretroviral treatment (ART) was introduced in Uganda in 1992 through a trial of zidovudine (AZT) by the Joint Clinical Research Center (JCRC). Subsequently, other ARVs were added as they became available. In 1998, the Ministry of Health (MoH), in collaboration with the Joint United Nations Programme on HIV & AIDS (UNAIDS), launched the Drug Access Initiative (DAI) project. ART is now operational in over 20 health facilities countrywide (17).

**Table 2.** Health characteristics of study participants (n=133) and household members

Variable	No.	%
Period since tested HIV-positive		
Less than one year	31	23.3
More than one year	97	73
Do not remember	5	3.7
Period taken to seek HIV care after diagnosis		
Immediately	96	72.2
Within one year	23	17.3
After one year	14	10.5
Taking ARVs		
Yes	24	18.0
No	109	82.0
Presence of other PLWHA in household		
Yes	48	36.1
No	36	27.1
Do not know	49	36.8
Distance (km) to the nearest health centre		
<2	97	72.9
>2	36	27.1

AIDS=Acquired immunodeficiency syndrome;

ARVs=Antiretrovirals;

HIV=Human immuno-deficiency virus;

PLWHA=People living with HIV and AIDS

In this study, 36.1% of the households had more than one person living with HIV and AIDS, thus a major concern for the increased burden of disease. Having more than one person living with HIV in household is known to create over-dependency as the livelihood opportunities of the households are likely to crumble.

### Nutritional knowledge, attitudes, and practices

#### Nutritional knowledge

Although 89.5% of the participants received training on nutrition, only 51.9% understood the meaning of good nutrition. Similarly, only 27.8% had knowledge of drug-food interactions (Table 3). Health workers/counsellors from HIV service organizations were the main source of health and nutrition information (89.9%). The information included general care for the PLWHA, importance of good nutrition for the PLWHA, and proper hygiene practices.

**Table 3.** Responses to nutritional KAP questions (n=133)

Variable	No.	%
Received training on nutrition and HIV	119	89.5
Correct definition of the term ‘good nutrition’	69	51.9
Knowledge of drug-food interactions	37	27.8
Importance and usefulness of the following for health and quality of life of PWLHA		
Consumption of a balanced diet	132	99.3
Consumption of fruits and vegetables	132	99.3
Increasing frequency of meal	60	45.1
Consumption of special diets	84	63.1
Number of meals consumed in the preceding 24 hours		
<3	104	78.2
≥3	29	21.8
Number of food-groups consumed in the preceding 24 hours		
<6 (inadequate)	80	60.2
≥6 (adequate)	53	39.8
Seasonal variations in diet	117	88.0
Consumption of special diets or supplements	27	20.3
Portioning meals within the household		
According to age-groups	97	72.9
According to health status	6	4.5
Practice of food taboos	10	7.5

AIDS=Acquired immunodeficiency syndrome;

HIV=Human immunodeficiency virus;

KAP=Knowledge, attitudes, practices;

PWLHA=People living with HIV and AIDS

#### Attitude on nutrition

Most participants understood that consumption of a balanced diet (99.3%), fruits and vegetables (99.3%), and special diets (63.1%) is necessary for good health. However, 45.1% believed that increasing the frequency of meal is important too.

#### Dietary practices

**Fig. 1.**
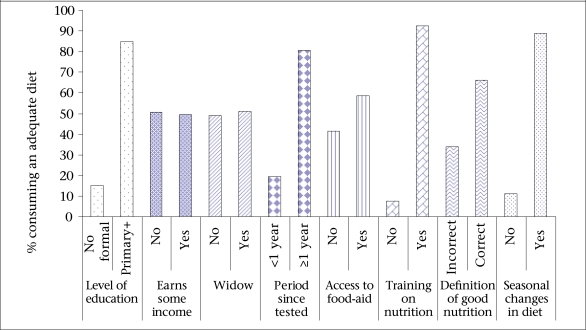
Factors associated with consumption of an adequate diet (≥6 food-groups)

Only 21.8% of the participants consumed three or more meals per day, and 39.8% consumed six or more food-groups in the preceding 24 hours to the interview. Seasonal variations in the number of meals and diversity of diet consumed in the households were, however, reported by most (88%) participants, with changes in prices of food likely to be the major contributing factor. Figure 1 shows that consumption of a diversified diet was significantly associated with the understanding of good nutrition (p=0.006) and meal frequency (p=0.002).

**Fig. 2.**
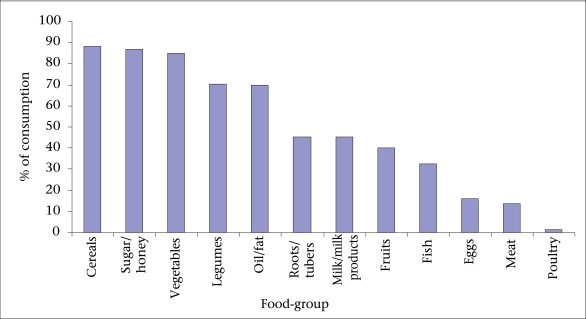
Consumption patterns of food-groups

Only 20.3% of the participants supplemented their regular household meals with special diets or nutrition supplements to meet the increased nutrition and energy needs of the PLWHA. In the large majority (72.9%) of the households, portioning of meals was according to age-group, and only 4.5% of the participants attributed it to health status; 7.5% of the participants reported food taboos. The participants’ higher dependency on starchy staples e.g. maize, rice, sweet potatoes, and cooked banana (*matooke*), was observed while foods of animal origin and fruits that play vital immunity and protective roles were poorly used (Fig. 2).

### Access to food-aid

Food-aid is mainly given to the most vulnerable, and possibly this explains why those who had been living with HIV for more than one year were more likely to be receiving food-aid than their counterparts (p<0.001), i.e. possible increased vulnerability in these households. These participants were also noted as most likely to consume at least three meals daily (p=0.036). In the absence of food-aid, the findings showed that the households were unable to ensure adequate dietary practices, particularly consumption of a diversified diet. Bivariate analysis indicated that consumption of a diversified diet was significantly associated with access to food-aid (p=0.006), possibly because access to food-aid reportedly enhances the ability of the household to access other food items (5).

## DISCUSSION

Food and nutrition security are fundamentally important for the prevention, care, treatment, and mitigation of HIV and AIDS. Gillespie and Kadiyala showed that a programme of care without a nutritional component is likely to crumble, and the efficacy of ART may be compromised by malnutrition (18). Similarly, since access to and availability of food are affected by the impact of HIV, any strategy to improve nutrition of those affected must prioritize enhancing appropriate nutritional knowledge, attitudes, and use of the little available food.

Poor nutritional knowledge, attitudes, and dietary practices, therefore, play a key role in the rapid progression of HIV. However, very little data exist concerning these aspects of nutrition among women living with HIV and AIDS in resource-limited settings, such as Uganda. These aspects are also among the key factors that determine the quality of life among PLWHA, although they have been largely overlooked, especially in resource-limited settings (19). Accordingly, the large majority (72.2%) of the study participants neither had the knowledge on important aspects of the role of nutrition in the enhancing treatment efficacy nor the drug-food interactions.

A high proportion (60.2%) of the participants reported consumption of less than six food-groups, implying a poor or an inadequate dietary quality as defined by Swindale and Ohri-Vachaspati (13). Lack of diversity in the diet is, therefore, a likely major contributory factor to inadequate intake of essential micronutrients. However, access to food-aid was a major boost for those households that received it. Poor dietary practices among the study population are an example of the negative coping strategies that HIV-affected households use as the HIV and AIDS pandemic steadily erodes positive coping mechanisms of the households over the long run. Results of a study showed that changes in dietary quantity and quality are among the common coping mechanisms in Uganda (5). The same study provided the evidence of negative impacts that HIV had on access to food in the affected households. The increasing failure of households to ensure the availability of adequate food and access, thus, hinders the practice of recommended nutrition habits for the PLWHA in these households. Other studies have shown low socioeconomic status, level of education, personal beliefs, availability of food, and low nutrition knowledge as contributory factors to poor dietary practices (16, 20–23).

The poor dietary practices among the PLWHA may also result from loss of appetite and anorexia, thus reducing the frequency of meal and variety at the very time when their requirements are higher, i.e. up to 50% and 15% increase in protein and energy respectively (24). Given the fact that the poor nutritional practices are linked to deterioration in immunity and subsequent nutritional status of the PLWHA, interventions geared at improving the practices are essential in the prevention of rapid progression of HIV.

The nutrition knowledge and positive attitudes gained by the PLWHA should be followed up to ensure that they are transformed into good dietary practices. Support groups of the PLWHA are a good vehicle for implementing these recommended practices and targeted nutrition interventions. Knowledge about drug-food interactions, use of fruits and vegetables as protective foods, and legumes as complementary protein source should be emphasized and promoted in the study area. Food-aid had a positive impact on dietary practices of beneficiary households. However, to ensure sustainable mitigation of the impacts of HIV and AIDS on affected households, it should be delivered as part of an integrated package of food security and nutrition programmes.

## ACKNOWLEDGEMENTS

The study was sponsored by the Nestle Foundation. The authors are grateful to the staff of the three HIV service organizations (TASO, JINNET+, and AOET) that played a key part in conducting the study.

## References

[1] Joint United Nations Programme on HIV & AIDS (2006). 2006 report on the global AID epidemic: A UNAIDS 10th anniversary special edition.

[2] Uganda (2005). Ministry of Health.

[3] Uganda AIDS Commission (2003). The HIV & AIDS epidemic: prevalence and impact.

[4] Moore DM, Hogg RS (2004). Trends in antenatal human immunodeficiency virus prevalence in Western Kenya and Eastern Uganda: evidence of differences in health policies?. Int J Epidemiol.

[5] Bukusuba J, Kikafunda JK, Whitehead RG (2007). Food security status in households of people living with HIV & AIDS (PLWHA) in a Ugandan urban setting. Br J Nutr.

[6] Tang AM, Graham NM, Kirby AJ, McCall LD, Willett WC, Saah AJ (1993). Dietary micronutrient intake and risk of progression to acquired immunodeficiency syndrome (AIDS) in human immunodeficiency virus type 1 (HIV-1)-infected homosexual men. Am J Epidemiol.

[7] Steinhart CR (2001). HIV-associated wasting in the era of highly active antiretroviral therapy (HAART): a practice-based approach to diagnosis and treatment. AIDS Reader.

[8] Tang AM (2003). Weight loss, wasting, and survival in HIV-positive patients: current strategies. AIDS.

[9] Walsh CM, Dannhauser A, Joubert G (2003). Impact of a nutrition education programme on nutrition knowledge and dietary practices of lower socioeconomic communities in the Free State and Northern Cape. SAJCN.

[10] Association of Nutrition Sciences Agencies (2002). Nutrition guidelines for agencies providing food to people living with HIV disease, 2nd ed..

[11] Swindale A, Bilinsky P (2005). Household dietary diversity score for measurement of household food access: indicator guide.

[12] Hoddinott J (1999). Choosing outcome indicators of household food security.

[13] Swindale A, Ohri-Vachaspati P (2004). Measuring household food consumption: a technical guide.

[14] Maxwell D, Levin C, Csete J (1998). Does urban agriculture help prevent malnutrition? Evidence from Kampala.

[15] Ruel MT (2002). Is dietary diversity an indicator of food security or dietary quality? A review of measurement issues and research needs.

[16] Torheim LE, Ouattara F, Diarra MM, Thiam FD, Barikmo I, HatlØy A (2004). Nutrient adequacy and dietary diversity in rural Mali: association and determinants. Eur J Clin Nutr.

[17] Uganda (2003). Ministry of Health. Antiretroviral treatment policy for Uganda.

[18] Gillespie S, Kadiyala S (2005). HIV & AIDS and food and nutrition security; from evidence to action.

[19] Kim JH, Spiegelman D, Rimm E, Gorbach SL (2001). The correlates of dietary intake among HIV-positive adults. Am J Clin Nutr.

[20] Hu SP, Liu JF, Shieh MJ (1997). Nutrition knowledge, attitudes and practices among senior medical students in Taiwan. J Am Coll Nutr.

[21] HatlØy A, Hallund J, Diarra MM, Oshaug A (2000). Food variety, socio-economic status and nutritional status in urban and rural areas in Koutiala (Mali). Public Health Nutr.

[22] Ogle BM, Hung PH, Tuyet HT (2001). Significance of wild vegetables in micronutrient intakes of women in Vietnam: an analysis of food variety. Asia Pac J Clin Nutr.

[23] Dallongeville J, Marecaux N, Cottel1 D, Bingham A, Amouyel P (2001). Association between nutrition knowledge and nutritional intake in middle-aged men from Northern France. Public Health Nutr.

[24] Haddad L, Gillespie S (2001). Effective food and nutrition policy responses to HIV & AIDS: what we know and what we need to know?.

